# Correlation between miR-200 Family Overexpression and Cancer Prognosis

**DOI:** 10.1155/2018/6071826

**Published:** 2018-07-04

**Authors:** Wen Liu, Kaiping Zhang, Pengfei Wei, Yue Hu, Yaqin Peng, Xiang Fang, Guoping He, Limin Wu, Min Chao, Jing Wang

**Affiliations:** ^1^Prenatal Diagnostic Center, Department of Obstetrics and Gynecology, The First Affiliated Hospital of University of Science and Technology of China, Anhui Provincial Hospital, Hefei, Anhui, China; ^2^Department of Urology, Anhui Provincial Children's Hospital and Children's Hospital of Anhui Medical University, Hefei, Anhui, China; ^3^Hefei National Laboratory for Physical Sciences at Microscale, CAS Key Laboratory of Innate Immunity and Chronic Disease, Innovation Center for Cell Signaling Network, School of Life Sciences and Medical Center, University of Science and Technology of China, Hefei, Anhui, China

## Abstract

The correlation between miR-200 family overexpression and cancer prognosis remains controversial. Therefore, we conducted a systematic review and meta-analysis by searching PubMed, Embase, Cochrane Library, China Biology Medicine disc (CBM), and China National Knowledge Infrastructure (CNKI) to identify eligible studies. Hazard ratios (HRs) with 95% confidence intervals (CIs) were calculated to evaluate the strength of the correlations. Additionally, different subgroup analyses and publication bias test were performed. Eventually, we analyzed 23 articles that included five tumor types and 3038 patients. Consequently, high expression of miR-200 family in various tumors was associated with unfavorable overall survival (OS) in both univariate (HR = 1.32, 95% CI: 1.14–1.54, *P* < 0.001) and multivariate (HR = 1.32, 95% CI: 1.16–1.49, *P* < 0.001) analyses. Likewise, a similar result was found in different subgroups of the patient source, cancer type, test method, sample source, miR-200 component, and sample size. However, no association of miR-200 family was detected with recurrence- or relapse-free survival (RFS) (univariate: HR = 1.02, 95% CI: 0.96–1.09, *P* = 0.47; multivariate: HR = 1.07, 95% CI: 1.00–1.14, *P* = 0.07), progression-free survival (PFS) (univariate: HR = 0.96, 95% CI: 0.54–1.70, *P* = 0.88; multivariate: HR = 1.17, 95% CI: 0.86–1.61, *P* = 0.32), and disease-free survival (DFS) (univariate: HR = 0.90, 95% CI: 0.74–1.09, *P* = 0.29; multivariate: HR = 0.98, 95% CI: 0.68–1.41, *P* = 0.90). Our findings have provided convincing evidence that miR-200 family overexpression suggested poor prognosis of various cancer types, which efforts may raise the potential use of miR-200 family for cancer prognosis in clinical practice.

## 1. Introduction

MicroRNAs (miRNAs) are evolutionarily conserved, endogenous small noncoding, and single-stranded RNAs of 18–22 nucleotides in length. They often negatively regulate gene targets by translational inhibition or mRNA degradation [1, 2]. It has been revealed that the posttranscriptional regulation could influence various biological processes including apoptosis, differentiation, proliferation, stress response, and metabolism [3, 4]. miRNAs could also be able to predict cancer prognosis due to their crucial roles in cancer progression and metastasis. Previous studies have explored that deregulated miRNAs with aberrant expression levels were closely correlated with cancer prognosis and even could be a novel kind of biomarkers for various cancer types [5, 6].

Interestingly, the miR-200 family is a typical and most extensively studied example in functional miRNAs. The miR-200 family, composed of five miRNA sequences (miR-141, miR-200a, miR-200b, miR-200c, and miR-429) and located in two clusters in the genome, is involved in the epithelial to mesenchymal transition (EMT) through regulation of E-cadherin expression via suppression of ZEB1 and ZEB2 [7, 8]. Recent studies have reported that miR-200 cluster is overexpressed in different tumors and played a critical role in mRNA degradation or inhibition through targeted binding to the relevant 3′-untranslated region (UTR) [9]. miR-200 family has been shown to offer a great potential in both cancer diagnosis and prognosis. Despite the potential roles of miR-200 family high expression in prognosis for cancer patients that have been attempted, no definite conclusions have been drawn so far. Meta-analysis can explore the authentic and comprehensive results through incorporating all available evidences to get a relatively precise and accurate estimation by using statistical analyses [10]. Thus, we have performed the current meta-analysis to explore the potential associations between miR-200 family and cancer prognosis, which efforts should hold great promise in verifying the potential of miRNAs as biomarkers for evaluating therapeutic efficacy and prognosis of various cancers.

## 2. Methods

### 2.1. Ethics Statement

The PRISMA statement was used to conduct the current meta-analysis [11]. No patient's privacy or clinical samples were involved in this study; hence, the ethical approval was not required.

### 2.2. Search Strategy

Literature resources including PubMed, Cochrane Library, Embase, CBM, and CNK were introduced to search eligible studies, by using the terms “microRNA OR miRNA OR miR-200 OR miR-141 OR miR-429 OR miR-200 family OR miR-200 cluster,” “survival OR prognosis OR prognostic,” and “cancer OR tumor OR tumour OR neoplasm OR neoplasma OR neoplasia OR carcinoma OR cancers OR tumors OR tumours OR neoplasms OR neoplasmas OR neoplasias OR carcinomas.” Last search of current investigation was updated on November 25th, 2017. Additionally, the publication language was only limited to English and Chinese. In case of omission, we identified the reference lists of the relevant articles and reviewed articles to seek for the potentially relevant studies. Conventionally, we have not contacted the corresponding authors even if the relevant data were unavailable.

### 2.3. Inclusion and Exclusion Criteria

Studies complied with the following criteria could be identified: (1) clinical study about the association of miR-200 family with cancer prognosis and (2) relevant data of the hazard ratios (HRs) and their corresponding 95% confidence intervals (CIs) to evaluate its associations were available. Studies which met the following four criteria were excluded: (1) the available data regarding associations was absent; (2) similar or duplicate study (when the same or similar cohort was applied, after careful examination, the most complete information was included); (3) other types of articles such as reviews or abstracts; and (4) studies involved with cell lines or animal models.

### 2.4. Data Extraction

In the light of inclusion and exclusion criteria, we extracted the relevant data from each eligible study. If disagreements were noticed, we are clearly open to discussion by each other (Wen Liu and Kaiping Zhang) or reviewed by a third author (Pengfei Wei). The data on first author, publication year, study country, age, cancer type, miRNA category, sample source, sample size, follow-up time, test method, survival outcome, analysis method, HR (95% CI), and the cut-off value were extracted. We have not contacted any author of the original researches even if the essential information could not be available. Besides, patient sources came from Asia, Europe, and North America. Sample sources were stratified into tissue, blood, formalin-fixed and paraffin-embedded (FFPE), and tissue microarray (TMA). Test methods included TaqMan, in situ hybridization (ISH), and reverse transcription polymerase chain reaction (RT-PCR). Sample sizes were separated into ≥100 and <100. Cancer types included epithelial ovarian cancer (EOC), breast cancer (BC), nonsmall cell lung cancer (NSCLC), gastric cancer (GC), and colorectal cancer (CRC). Analyses methods were divided into univariate analysis and multivariate analysis. Patients' prognostic outcomes included overall survival (OS), relapse-free survival (RFS), progression-free survival (PFS), and disease-free survival (DFS).

## 3. Statistical Analysis

We have explored the association of miR-200 family with cancer prognosis by applying Review Manager software (RevMan 5, The Cochrane Collaboration, Oxford, UK) and Stata software (Version 12.0, Stata Corporation, College Station, TX). HR and 95% CI were collected for assessing the prognostic value of high expression of miR-200 family in various cancers. Meanwhile, the heterogeneity has been assessed via chi-square-based *Q* and *I*^2^ test across studies (no heterogeneity *I*^2^ < 25%, moderate heterogeneity *I*^2^ = 25%–50%, extreme heterogeneity *I*^2^ > 50%) [12]. In case of extreme heterogeneity (*I*^2^ > 50% or *P* < 0.01 for *Q* test), we used random-effects (DerSimonian and Laird method) model [13]. Otherwise, fixed-effects (Mantel-Haenszel method) model was introduced [14]. One-way sensitivity analyses which individually removed publications in meta-analysis were conducted to assess results' stability. It mainly explores the impact of specific study upon mixed HR. In Begg's funnel plots, logHR was plotted against SE. *P* value less than 0.05 indicated that there was a bias of the study [15]. Additionally, different subgroups consisted of patient source, cancer type, test method, sample source, sample size, and miR-200 component were conducted.

## 4. Results

### 4.1. Characteristics of the Studies

**Figure 1:**
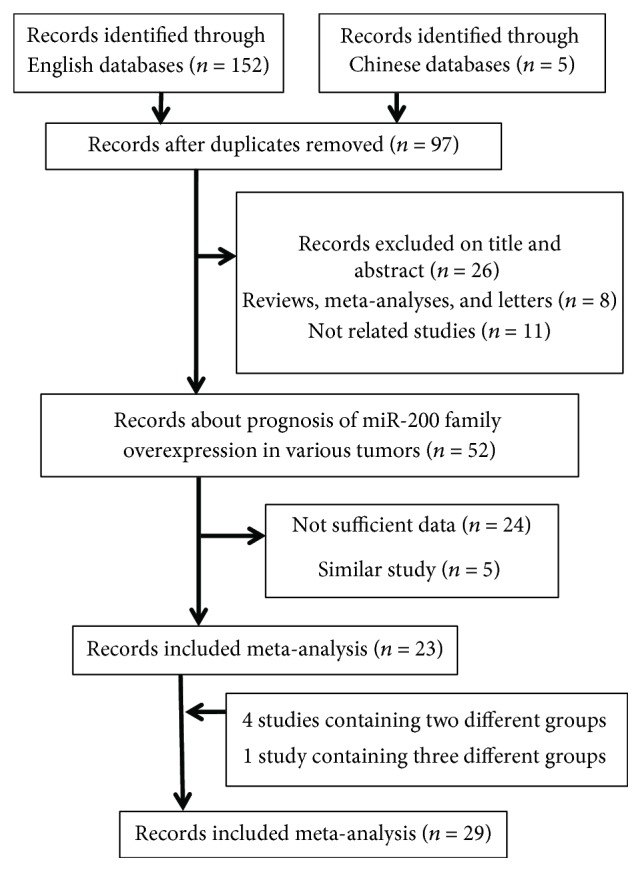
Flow diagram of the study selection process in the meta-analysis.

Consequently, 23 studies consisted of 3038 samples satisfied the eligible criteria [16–38] (Figure 1).

The principal characteristics of the eligible studies were summarized in Table 1.

Among these studies, Cheng's study was involved with three different cohorts of Tianjin cohort, TexGen cohort, and all cohort [36]. Zhu et al. designed a study to detect tissue and serum miRNA expression [28]. Tejero et al. analyzed the role of members of the miR-200 family from NSCLC patients after surgery both in the entire cohort and adenocarcinoma cohort [30]. Maierthaler et al. explore miRNA expression in two different cohorts of nonmetastatic and metastatic CRC [18]. Toiyama et al. conducted a study to detect the prognostic value of the miR-200 family in CRC from blood and FFPE samples. As mentioned above, we treated them independently into meta-analysis [31]. Eventually, this meta-analysis was established based on 29 studies (Table 2). Among these 29 studies, 28 were written in English while one was published in Chinese. The sample sizes ranged from 44 to 527. The cancer types contained ten EOC, one BC, seven NSCLC, two GC, and nine CRC. Meanwhile, one ISH, 24 RT-PCR, and four TaqMan in test methods were applied. According to the sample sources, there were seven FFPE, ten tissue, ten blood, and two TMA. For the survival outcomes, 29 eligible studies were divided into 42 datasets: 29 for OS, six for PFS, five for RFS, and two for DFS. However, the cut-off value for the miR-200 family was inconsistent among these included studies (Table 2).

### 4.2. Meta-Analysis of OS

**Figure 2:**
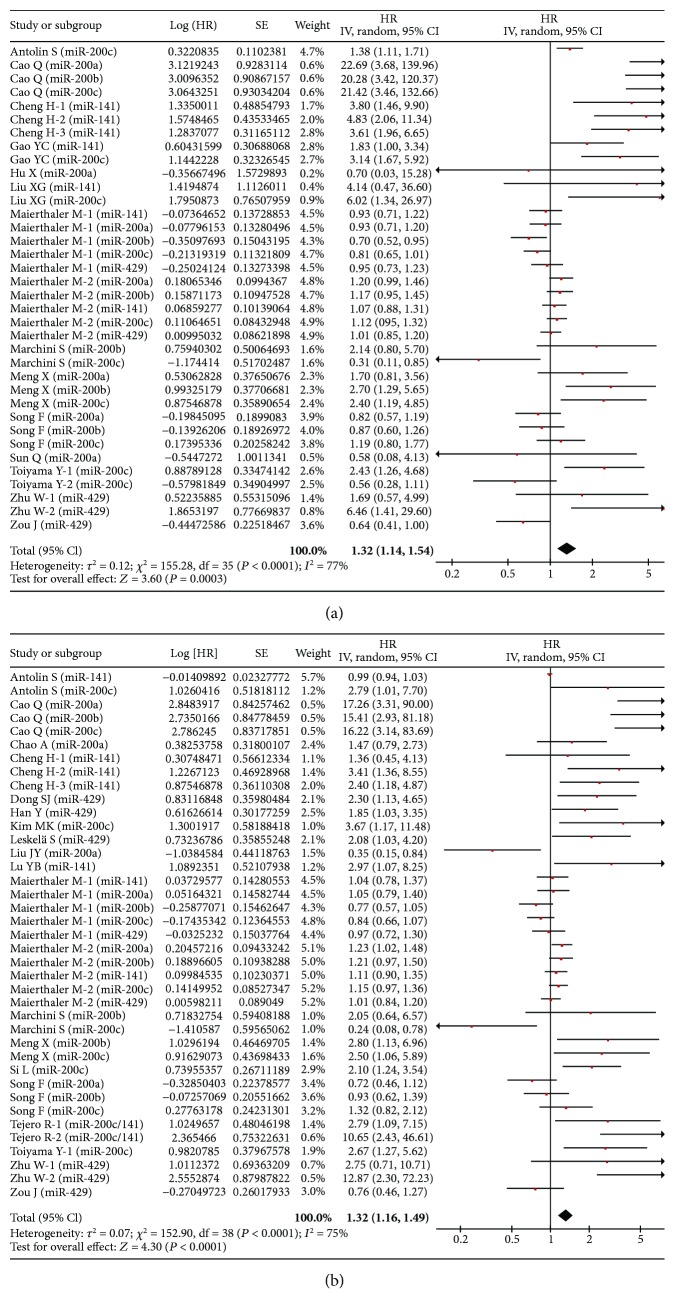
Forest plot of the association between high expression of the miR-200 family in various tumors and OS under different types of analysis. (a) Univariate analysis; (b) multivariate analysis. The squares and horizontal lines correspond to the study-specific HR and 95% CI. The area of the squares reflects the weight. The diamond represents the summary HR and 95% CI. CI = confidence interval, HR = hazard ratio.

In univariate analysis, 19 studies were involved in current meta-analysis to assess the prognosis of miR-200 family overexpression in various cancers. High expression of miR-200 family was found to be associated with unfavorable OS (HR = 1.32, 95% CI: 1.14–1.54, *P* < 0.001) (Figure 2(a)). Besides, it indicated that there were certain associations via subanalyses regarding patient source, cancer type, test method, sample source, sample size, and miR-200 component (Table 3).

In multivariate analysis, 24 studies were included in meta-analysis to explore the prognostic value of the miR-200 family. As a result, high expression of the miR-200 family in various cancers was associated with unfavorable overall survival (HR = 1.32, 95% CI: 1.16–1.49, *P* < 0.001) (Figure 2(b)). Likewise, a similar result was found in different subgroups (Table 3).

### 4.3. Meta-Analysis of RFS/PFS/DFS

**Figure 3:**
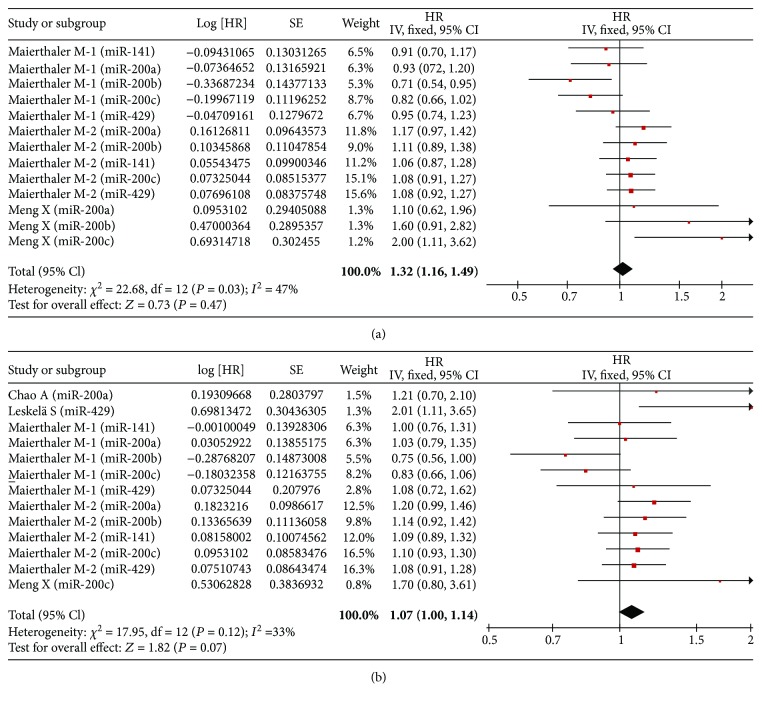
Forest plot of the association between high expression of the miR-200 family in various tumors and RFS under different types of analysis. (a) Univariate analysis; (b) multivariate analysis. The squares and horizontal lines correspond to the study-specific HR and 95% CI. The area of the squares reflects the weight. The diamond represents the summary HR and 95% CI. CI = confidence interval, HR = hazard ratio.

**Figure 4:**
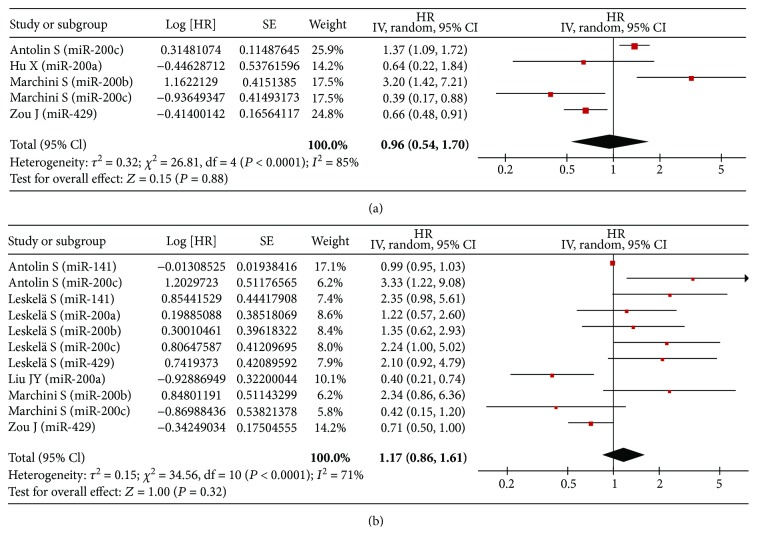
Forest plot of the association between high expression of the miR-200 family in various tumors and PFS under different types of analysis. (a) Univariate analysis; (b) multivariate analysis. The squares and horizontal lines correspond to the study-specific HR and 95% CI. The area of the squares reflects the weight. The diamond represents the summary HR and 95% CI. CI = confidence interval, HR = hazard ratio.

**Figure 5:**
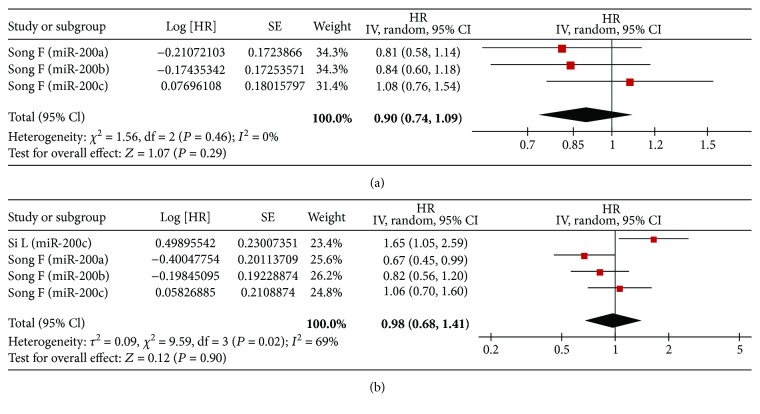
Forest plot of the association between high expression of the miR-200 family in various tumors and DFS under different types of analysis. (a) Univariate analysis; (b) multivariate analysis. The squares and horizontal lines correspond to the study-specific HR and 95% CI. The area of the squares reflects the weight. The diamond represents the summary HR and 95% CI. CI = confidence interval, HR = hazard ratio.

In univariate analysis, there were three studies, four studies, and one study involved with RFS, PFS, and DFS, respectively. Correspondingly, five studies, five studies, and two studies were collected in multivariate analysis, respectively. Ultimately, we found that no association of high expression of the miR-200 family was detected with RFS (univariate: HR = 1.02, 95% CI: 0.96–1.09, *P* = 0.47; multivariate: HR = 1.07, 95% CI: 1.00–1.14, *P* = 0.07) (Figure 3), PFS (univariate: HR = 0.96, 95% CI: 0.54–1.70, *P* = 0.88; multivariate: HR = 1.17, 95% CI: 0.86–1.61, *P* = 0.32) (Figure 4), and DFS (univariate: HR = 0.90, 95% CI: 0.74–1.09, *P* = 0.29; multivariate: HR = 0.98, 95% CI: 0.68–1.41, *P* = 0.90) (Figure 5).

### 4.4. Sensitivity Analysis

**Figure 6:**
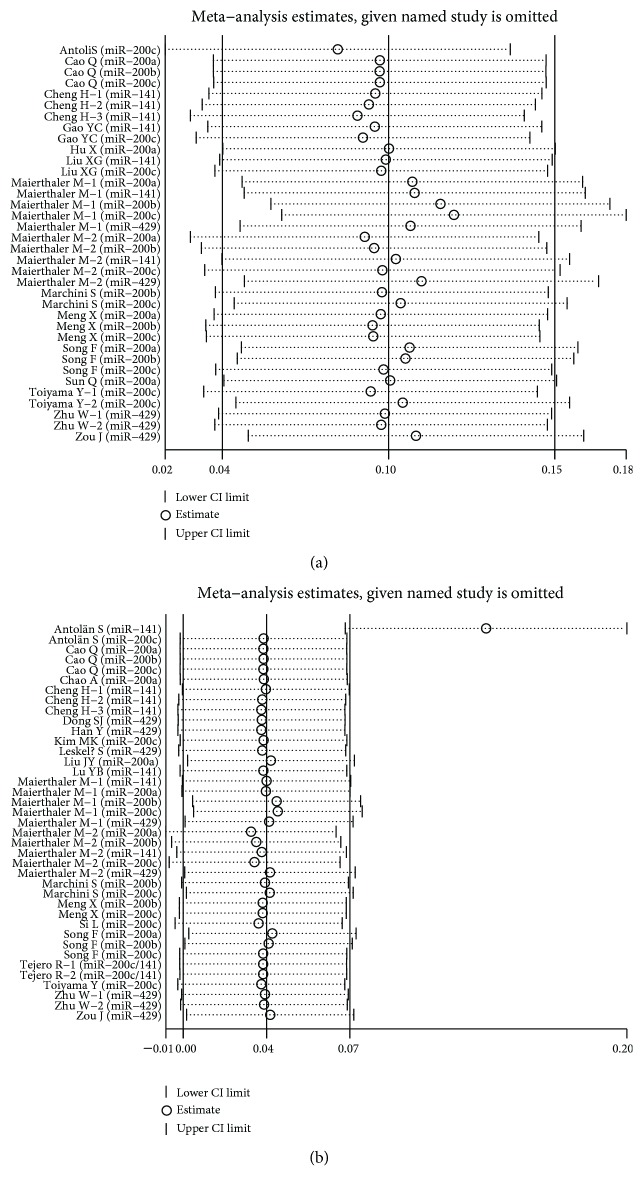
One-way sensitivity analysis of high expression of the miR-200 family in various tumors with OS under different types of analysis. (a) Univariate analysis; (b) multivariate analysis. Individually removed the studies and suggested that the results of this meta-analysis were relatively stable.

**Figure 7:**
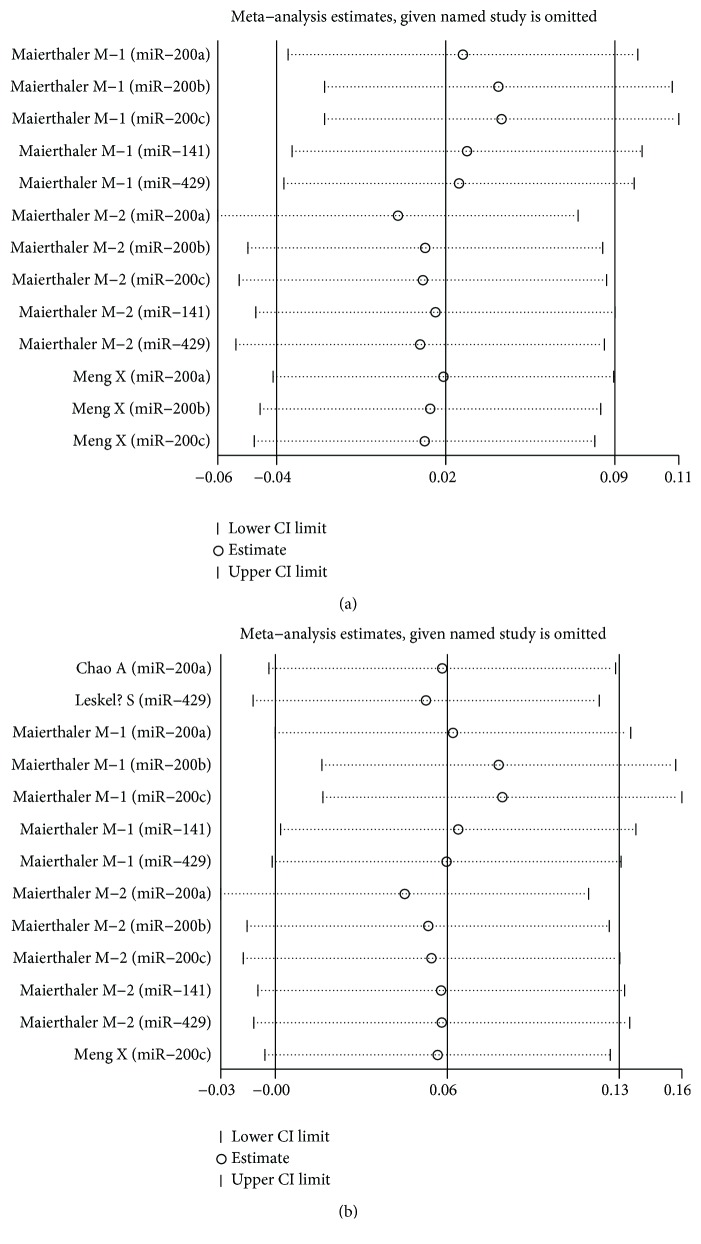
One-way sensitivity analysis of high expression of the miR-200 family in various tumors with RFS under different types of analysis. (a) Univariate analysis; (b) multivariate analysis. Individually removed the studies and suggested that the results of this meta-analysis were stable.

**Figure 8:**
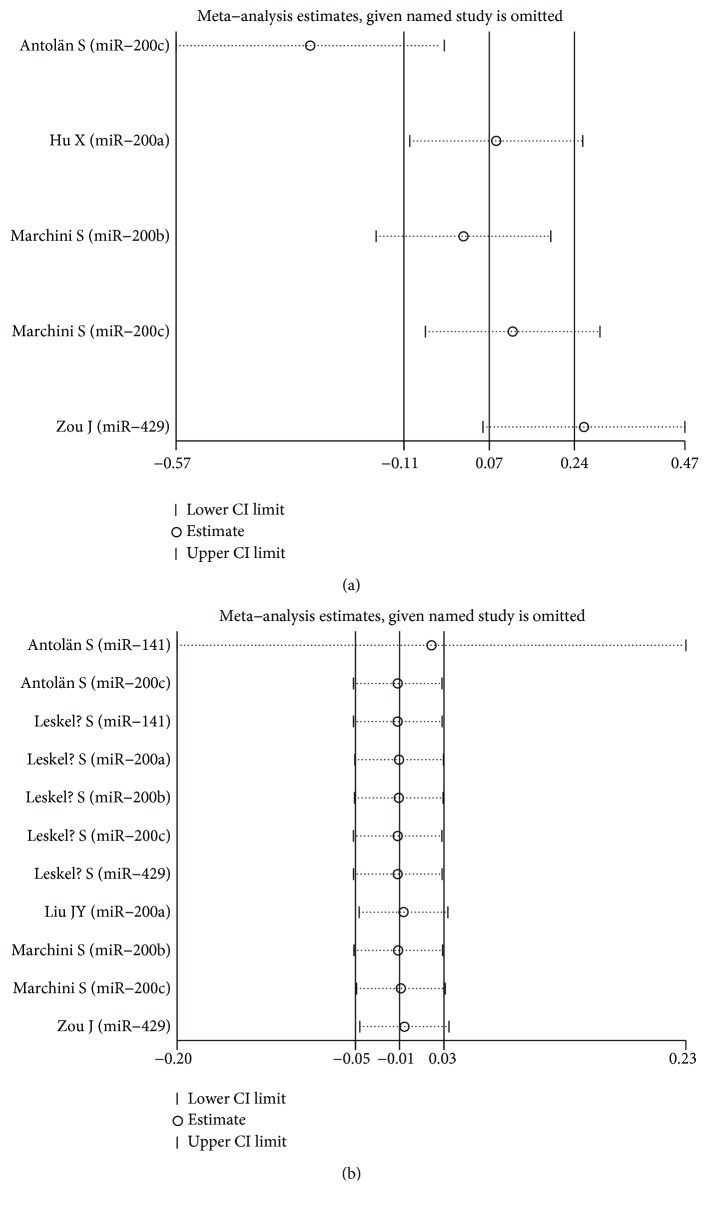
One-way sensitivity analysis of high expression of the miR-200 family in various tumors with PFS under different types of analysis. (a) Univariate analysis; (b) multivariate analysis. Individually removed the studies and suggested that the results of this meta-analysis were relatively stable.

Each single included study was deleted at a time to assess the specific effect of the individual data on the pooled HRs, and one-way sensitivity analysis suggested that most pooled results were relatively stable. Among them, the pooled results of OS, RFS, and PFS in both univariate analysis and multivariate analysis were shown in Figures 6(a), 6(b), Figures 7(a), 7(b), and Figures 8(a), 8(b), respectively. As shown in Figure 6(b), after excluding the study conducted by Antolín et al. [22], heterogeneity was slightly reduced between miR-200 family overexpression and OS under multivariate analysis (*I*^2^ from 75.1% to 73.3%), while the pooled results remained unchanged (multivariate: HR = 1.40, 95% CI: 1.21–1.63, *P* < 0.001). Likewise, as shown in Figure 8(a), the similar result was found between miR-200 family overexpression and PFS under univariate analysis (*I*^2^ from 85.1% to 80.4%), and the pooled results remained unchanged (univariate: HR = 0.85, 95% CI: 0.38–1.88, *P* = 0.684) after excluding the aforementioned study [22].

### 4.5. Publication Bias Evaluation

Begg's funnel plot indicated that there was a significant publication bias in meta-analysis of OS under both univariate analysis (*P* = 0.028) and multivariate analysis (*P* < 0.001). However, no publication bias was found in meta-analysis of RFS (univariate: *P* = 0.760; multivariate: *P* = 0.855), PFS (univariate: *P* = 1.000; multivariate: *P* = 0.087), and DFS (univariate: *P* = 0.296; multivariate: *P* = 0.308).

## 5. Discussion

Generally, cancer progression and blood-borne metastasis are the primary factors contributed to the great majority of cancer deaths. The specific biomarkers of metastatic phenotype hold great promise in individualized therapy and improved prognosis prediction in several neoplastic diseases [39]. In recent decades, to explore the clinically useful cancer signatures remains to be research hotpot due to the complexity of cancer. Gene expression signatures of carcinomas have led to new classifications of cancer subgroups and also carried prognostic and predictive information [40]. miRNAs are small noncoding RNAs that regulate human protein-coding gene expression of specific mRNAs by either translational repression or degradation. miRNA expression signatures have distinct functions in controlling the cell cycle, proliferation, invasion, and metastasis [41], which could thus be developed into a potential prognostic signature [42]. The latest miRBase release contains 24,521 miRNA loci from 206 species, further processed to produce 30,424 mature miRNA products [43]. To date, significant miRNA expression changes have been observed in multiple cancers analyzed by profiling and next generation sequencing technologies [44].

The miR-200 family of miRNAs consists of five members grouped into two independent transcriptional clusters: miR-200a, miR-200b, and miR-429 on chromosome 1 (1p36.33), and miR-141 and miR-200c on chromosome 12 (12p13.31). Deregulation of the miR-200 family of microRNAs has been involved in cell plasticity, apoptosis, molecular subtype, oestrogen regulation, control of the growth and function of stem cells, and regulation of the downstream transcriptional program that mediate distant metastasis [45]. Cancer progression is associated with a dynamic process of epithelial-to-mesenchymal transition (EMT), during which epithelial cells lose their cell polarity and cell-cell adhesion and gain migratory as well as invasive properties by downregulating E-cadherin and upregulating vimentin expression [46, 47]. The miR-200 family members may play a major role in the suppression of EMT and metastasis [48]. Deregulation of miR-200 in cancer cell lines caused upregulation of E-cadherin and reduced motility of cancer cells. Conversely, inhibition of miR-200 reduced E-cadherin expression, increased expression of vimentin, and induced EMT [49]. In addition, the miR-200 family is known as a key transcriptional regulator of EMT and the maintenance of a less invasive and aggressive epithelial phenotype by targeting ZEB1 and ZEB2, two important transcriptional repressors of the E-cadherin gene [48]. ZEB was inhibited by miR-200 members at the posttranscriptional level by binding to highly conserved target sites in their 3′-UTR; the functional link of ZEB factors with the miR-200 family in a double negative feedback loop is known as the ZEB/miR-200 feedback loop [50]. It also has been reported that several tumor suppressor genes, including BRD7, BAP1, GATA, CLOCK, and PTPN12, might be potential targets of the miR-200 family [51, 52].

To date, studies focused on the association of high expression of the miR-200 family with cancer prognosis have yielded conflicting results. Notably, small sample-sized studies lacking statistical power often have resulted in apparently contradicting conclusions. Meta-analysis is a useful tool for providing convincing evidence as it could present inconsistent results from different studies to get a relatively precise result. As far as we know, the current meta-analysis is the first try to comprehensively assess the correlation of miR-200 cluster high expression with cancer prognosis. We have explored the potential associations in overall population and the corresponding subgroups. Consequently, of particular interest is the finding of significant correlation between high expression of miR-200 cluster and poor OS by two different statistical methods. Likewise, a similar result was found in different subgroups. However, no association of miR-200 family was detected with RFS/PFS/DFS.

In the current meta-analysis, significant heterogeneity was found, which required careful interpretation and searched for influencing factors by further subgroup analyses. Consequently, impact of ethnicity, detection methods, cancer types, sample size, and sample source on prognosis in patients was considerable, which should be taken into consideration when evaluating the prognosis of cancer for patients. Some potential or undiscovered factors including adjustment for surgery, radiation, chemotherapy, socioeconomic status, and tumor characteristics should not be ignored. Moreover, there was a significant publication bias in meta-analysis of OS under both univariate analysis and multivariate analysis, suggesting that only published studies in English and Chinese might not provide so sufficient evidences. As for RFS/PFS/DFS, we did not perform subgroup analyses due to relatively fewer eligible studies. Although the studies regarding various tumors without a consistent cut-off value may influence the ultimate results and the heterogeneity suggested that potential or undiscovered factors might be ignored, a certain relationship of high expression of the miR-200 family in cancer prognosis was found in the current study.

## 6. Conclusion

In summary, the current study is the first original meta-analysis to address the correlation between miR-200 family expression and prognosis for cancer patients. A significant correlation was explored in overall population as well as the corresponding subgroups. Concretely, it presented that miR-200 family overexpression might be associated with poor OS to some extent, while no association was detected between high miR-200 family expression and RFS/PFS/DFS. In the future, detailed investigations comprising large cohort size from multicenter are required to confirm our conclusions.

## Figures and Tables

**Table 1:** Main characteristics of the eligible studies.

First author	Year	Country	Age	Cancer type	MicroRNA	Sample size	Follow-up, median (range)	Outcome
Zou J. [16]	2017	China	NA	EOC	miR-429	72	NA	OS/PFS
Han Y. [17]	2017	China	NA	CRC	miR-429	71	34.2	OS
Maierthaler M. [18]	2017	Germany	70 (33–92) 68.0 (36–92)	CRC	miR-200a, miR-200b, miR-200c, miR-141, miR-429	527	NA	OS/RFS
Si L. [19]	2017	China	60.5 (41–78)	NSCLC	miR-200c	110	NA	OS/DFS
Meng X. [20]	2016	Germany	60 (23–91)	EOC	miR-200a, miR-200b, miR-200c	163	20 (1–136)	OS/RFS
Dong S. J. [21]	2016	China	56 (31–79)	CRC	miR-429	116	NA	OS
Antolín S. [22]	2015	Spain	54.8 (29–73)	BC	miR-200c, miR-141	57	74.6 (74.2–77.7)	OS/PFS
Gao Y. C. [23]	2015	China	NA	EOC	miR-200c, miR-141	93	NA	OS
Lu Y. B. [24]	2015	China	NA	GC	miR-141	95	NA	OS
Liu J. Y. [25]	2015	China	57.48	EOC	miR-200a	44	26 (5–49)	OS/PFS
Cao Q. [26]	2014	China	58 (26–88)	EOC	miR-200a, miR-200b, miR-200c	100	36.8 (6–56)	OS
Kim M. K. [27]	2014	Korea	64 (26–77)	NSCLC	miR-200c	72	31 (1–135)	OS
Zhu W. [28]	2014	China	59	NSCLC	miR-429	70	NA	OS
Song F. [29]	2014	China	60.5	GC	miR-200a, miR-200b, miR-200c	385	35 (1–112)	OS/PFS
Tejero R. [30]	2014	Spain	65 (35–85)	NSCLC	miR-200c/141	155	43 (2–160)	OS
Toiyama Y. [31]	2014	Japan	67	CRC	miR-200c	182	NA	OS
Sun Q. [32]	2014	China	NA	EOC	miR-200a	53	56.79 (11–98)	OS
Liu X. G. [33]	2012	China	NA	NSCLC	miR-200c, miR-141	70	24	OS
Chao A. [34]	2012	China	NA	EOC	miR-200a	176	40 (3–109)	OS/RFS
Marchini S. [35]	2011	Italy	52 (21–82)	EOC	miR-200b, miR-200c	144	110.4 (82.8–139.2)	OS/PFS
Cheng H. [36]	2011	USA	NA	CRC	miR-141	156	NA	OS
Leskelä S. [37]	2010	Spain	57 (35–85)	EOC	miR-200a, miR-200b, miR-200c, miR-141, miR-429	72	NA	OS/PFS/RFS
Hu X. [38]	2009	USA	58.3	EOC	miR-200a	55	NA	OS/PFS

NA: not available; EOC: epithelial ovarian cancer; BC: breast cancer; NSCLC: nonsmall cell lung cancer; GC: gastric cancer; CRC: colorectal cancer; OS: overall survival; DFS: disease-free survival; PFS: progression-free survival; RFS: recurrence- or relapse-free survival; HR: hazard ratio; CI: confidence interval.

**Table 2:** MicroRNA evaluation and survival data of the selected studies.

First author	Year	Country	Test method	Cancer type	MicroRNA	Sample source	Outcome	HR (95% CI)	Cut-off value
Zou J.	2017	China	RT-PCR	EOC	miR-429	Tissue	OS	(U) 0.641 (0.412–0.996)/(M) 0.763 (0.458–1.270)	>0.532
Zou J.	2017	China	RT-PCR	EOC	miR-429	Tissue	PFS	(U) 0.661 (0.478–0.915)/(M) 0.710 (0.504–1.001)	

Han Y.	2017	China	RT-PCR	CRC	miR-429	Tissue	OS	(M) 1.852 (1.019–3.326)	Median

Maierthaler M.-1	2017	Germany	TaqMan	CRC	miR-200a	Blood	OS	(U) 0.929 (0.707–1.211)/(M) 1.053 (0.791–1.401)	Median
Maierthaler M.-1	2017	Germany	TaqMan	CRC	miR-200b	Blood	OS	(U) 0.704 (0.524–0.945)/(M) 0.772 (0.570–1.045)	
Maierthaler M.-1	2017	Germany	TaqMan	CRC	miR-200c	Blood	OS	(U) 0.808 (0.646–1.010)/(M) 0.840 (0.659–1.070)	
Maierthaler M.-1	2017	Germany	TaqMan	CRC	miR-141	Blood	OS	(U) 0.925 (0.713–1.200)/(M) 1.038 (0.785–1.374)	
Maierthaler M.-1	2017	Germany	TaqMan	CRC	miR-429	Blood	OS	(U) 0.951 (0.734–1.235)/(M) 0.968 (0.721–1.300)	
Maierthaler M.-2	2017	Germany	TaqMan	CRC	miR-200a	Blood	OS	(U) 1.198 (0.986–1.456)/(M) 1.227 (1.008–1.495)	
Maierthaler M.-2	2017	Germany	TaqMan	CRC	miR-200b	Blood	OS	(U) 1.172 (0.946–1.453)/(M) 1.208 (0.975–1.497)	
Maierthaler M.-2	2017	Germany	TaqMan	CRC	miR-200c	Blood	OS	(U) 1.117 (0.947–1.318)/(M) 1.152 (0.975–1.362)	
Maierthaler M.-2	2017	Germany	TaqMan	CRC	miR-141	Blood	OS	(U) 1.071 (0.877–1.305)/(M) 1.105 (0.904–1.350)	
Maierthaler M.-2	2017	Germany	TaqMan	CRC	miR-429	Blood	OS	(U) 1.010 (0.853–1.196)/(M) 1.006 (0.845–1.198)	
Maierthaler M.-1	2017	Germany	TaqMan	CRC	miR-200a	Blood	RFS	(U) 0.929 (0.718–1.203)/(M) 1.031 (0.786–1.353)	
Maierthaler M.-1	2017	Germany	TaqMan	CRC	miR-200b	Blood	RFS	(U) 0.714 (0.539–0.947)/(M) 0.750 (0.561–1.005)	
Maierthaler M.-1	2017	Germany	TaqMan	CRC	miR-200c	Blood	RFS	(U) 0.819 (0.657–1.019)/(M) 0.835 (0.658–1.060)	
Maierthaler M.-1	2017	Germany	TaqMan	CRC	miR-141	Blood	RFS	(U) 0.910 (0.705–1.175)/(M) 0.999 (0.760–1.312)	
Maierthaler M.-1	2017	Germany	TaqMan	CRC	miR-429	Blood	RFS	(U) 0.954 (0.743–1.227)/(M) 1.076 (0.716–1.618)	
Maierthaler M.-2	2017	Germany	TaqMan	CRC	miR-200a	Blood	RFS	(U) 1.175 (0.973–1.420)/(M) 1.200 (0.989–1.456)	
Maierthaler M.-2	2017	Germany	TaqMan	CRC	miR-200b	Blood	RFS	(U) 1.109 (0.893–1.377)/(M) 1.143 (0.919–1.422)	
Maierthaler M.-2	2017	Germany	TaqMan	CRC	miR-200c	Blood	RFS	(U) 1.076 (0.911–1.272)/(M) 1.100 (0.930–1.302)	
Maierthaler M.-2	2017	Germany	TaqMan	CRC	miR-141	Blood	RFS	(U) 1.057 (0.871–1.284)/(M) 1.085 (0.890–1.321)	
Maierthaler M.-2	2017	Germany	TaqMan	CRC	miR-429	Blood	RFS	(U) 1.080 (0.916–1.272)/(M) 1.078 (0.910–1.277)	

Si L.	2017	China	RT-PCR	NSCLC	miR-200c	Tissue	OS	(M) 2.095 (1.241–3.536)	The 2−ΔΔCq
Si L.	2017	China	RT-PCR	NSCLC	miR-200c	Tissue	DFS	(M) 1.647 (1.049–2.585)	

Meng X.	2016	Germany	RT-PCR	EOC	miR-200a	Blood	OS	(U) 1.7 (0.8–3.5)	Median
Meng X.	2016	Germany	RT-PCR	EOC	miR-200b	Blood	OS	(U) 2.7 (1.3–5.7)/(M) 2.8 (1.1–6.8)	
Meng X.	2016	Germany	RT-PCR	EOC	miR-200c	Blood	OS	(U) 2.4 (1.2–4.9)/(M) 2.5 (1.1–6.1)	
Meng X.	2016	Germany	RT-PCR	EOC	miR-200a	Blood	RFS	(U) 1.1 (0.6–1.9)	
Meng X.	2016	Germany	RT-PCR	EOC	miR-200b	Blood	RFS	(U) 1.6 (0.9–2.8)	
Meng X	2016	Germany	RT-PCR	EOC	miR-200c	Blood	RFS	(U) 2.0 (1.1–3.6)/(M) 1.7 (0.8–3.6)	

Dong S. J.	2016	China	RT-PCR	CRC	miR-429	Tissue	OS	(M) 2.296 (1.105–4.528)	Median

Antolín S.	2015	Spain	RT-PCR	BC	miR-200c	Blood	OS	(U) 1.38 (1.11–1.71)/(M) 2.79 (1.01–7.7)	>1.29 relative expression value
Antolín S.	2015	Spain	RT-PCR	BC	miR-200c	Blood	PFS	(U) 1.37 (1.09–1.71)/(M) 3.33 (1.22–9.07)	
Antolín S.	2015	Spain	RT-PCR	BC	miR-141	Blood	OS	(M) 0.986 (0.942–1.032)	
Antolín S.	2015	Spain	RT-PCR	BC	miR-141	Blood	PFS	(M) 0.987 (0.95–1.025)	

Gao Y. C.	2015	China	RT-PCR	EOC	miR-200c	Blood	OS	(U) 3.14 (1.67–5.93)	−ΔCt method with 95% CI
Gao Y. C.	2015	China	RT-PCR	EOC	miR-141	Blood	OS	(U) 1.83 (1.00–3.33)	

Lu Y. B.	2015	China	RT-PCR	GC	miR-141	Tissue	OS	(M) 2.972 (1.297–10.001)	Median

Liu J. Y.	2015	China	RT-PCR	EOC	miR-200a	Tissue	OS	(M) 0.354 (0.149–0.840)	Log 2−ΔΔCt
Liu J. Y.	2015	China	RT-PCR	EOC	miR-200a	Tissue	PFS	(M) 0.395 (0.210–0.742)	

Cao Q	2014	China	ISH	EOC	miR-200a	Tissue	OS	(U) 22.69 (1.32–50.53)/(M) 17.26 (1.36–36.98)	Median
Cao Q.	2014	China	ISH	EOC	miR-200b	Tissue	OS	(U) 20.28 (1.20–42.28)/(M)15.41 (1.13–31.36)	
Cao Q.	2014	China	ISH	EOC	miR-200c	Tissue	OS	(U) 21.42 (1.26–48.33)/(M) 16.22 (1.27–33.81)	

Kim M. K.	2014	Korea	RT-PCR	NSCLC	miR-200c	FFPE	OS	(M) 3.67 (1.17–11.45)	Median

Zhu W.-1	2014	China	RT-PCR	NSCLC	miR-429	Tissue	OS	(U) 1.686 (0.570–4.984)/(M) 2.749 (0.706–10.707)	Mean
Zhu W.-2	2014	China	RT-PCR	NSCLC	miR-429	Blood	OS	(U) 6.458 (1.409–29.593)/(M) 12.875 (2.295–72.23)	

Song F.	2014	China	RT-PCR	GC	miR-200a	TMA	OS	(U) 0.82 (0.57–1.20)/(M) 0.72 (0.47–1.13)	Median
Song F.	2014	China	RT-PCR	GC	miR-200b	TMA	OS	(U) 0.87 (0.60–1.26)/(M)0.93 (0.63–1.41)	
Song F.	2014	China	RT-PCR	GC	miR-200c	TMA	OS	(U) 1.19 (0.80–1.77)/(M) 1.32 (0.82–2.12)	
Song F.	2014	China	RT-PCR	GC	miR-200a	TMA	DFS	(U) 0.81 (0.58–1.14)/(M) 0.67 (0.45–0.99)	
Song F.	2014	China	RT-PCR	GC	miR-200b	TMA	DFS	(U) 0.84 (0.60–1.18)/(M) 0.82 (0.56–1.19)	
Song F.	2014	China	RT-PCR	GC	miR-200c	TMA	DFS	(U) 1.08 (0.76–1.54)/(M) 1.06 (0.70–1.60)	

Tejero R.-1	2014	Spain	TaqMan	NSCLC	miR-200c/141	FFPE	OS	(M) 2.787 (1.087–7.148)	Mean
Tejero R.-2	2014	Spain	TaqMan	NSCLC	miR-200c/141	FFPE	OS	(M) 10.649 (2.433–46.608)	

Toiyama Y.-1	2014	Japan	RT-PCR	CRC	miR-200c	Blood	OS	(U) 2.43 (1.26–4.68)/(M)2.67 (1.28–5.67)	Median
Toiyama Y.-2	2014	Japan	RT-PCR	CRC	miR-200c	FFPE	OS	(U) 0.56 (0.28–1.10)	

Sun Q.	2014	China	RT-PCR	EOC	miR-200a	TMA	OS	(U) 0.58 (0.08–4.05)	Median (≥12.623)

Liu X. G.	2012	China	RT-PCR	NSCLC	miR-200c	Tissue	OS	(U) 6.020 (1.344–26.971)	2−ΔΔCt > 2.0
Liu X. G.	2012	China	RT-PCR	NSCLC	miR-141	Tissue	OS	(U) 4.135 (0.467–36.597)	
Chao A.	2012	China	RT-PCR	EOC	miR-200a	FFPE	OS	(M) 1.466 (0.786–2.734)	Log ratio > 1.3
Chao A.	2012	China	RT-PCR	EOC	miR-200a	FFPE	RFS	(M) 1.213 (0.70–2.101)	

Marchini S.	2011	Italy	RT-PCR	EOC	miR-200b	Tissue	OS	(U) 2.137 (0.801–5.701)/(M) 2.051 (0.640–6.570)	>25%
Marchini S.	2011	Italy	RT-PCR	EOC	miR-200b	Tissue	PFS	(U) 3.197 (1.417–7.213)/(M) 2.335 (0.857–6.363)	
Marchini S.	2011	Italy	RT-PCR	EOC	miR-200c	Tissue	OS	(U) 0.309 (0.112–0.850)/(M) 0.244 (0.076–0.785)	
Marchini S.	2011	Italy	RT-PCR	EOC	miR-200c	Tissue	PFS	(U) 0.392 (0.174–0.885)/(M) 0.419 (0.146–1.204)	

Cheng H.-1	2011	USA	RT-PCR	CRC	miR-141	Blood	OS	(U) 3.80 (1.46–9.91)/(M) 1.36 (0.45–4.14)	2−ΔΔCt
Cheng H.-2	2011	USA	RT-PCR	CRC	miR-141	Blood	OS	(U) 4.83 (2.06–11.35)/(M) 3.41 (1.36–8.56)	
Cheng H.-3	2011	USA	RT-PCR	CRC	miR-141	Blood	OS	(U) 3.61 (1.96–6.65)/(M) 2.40 (1.18–4.86)	

Leskelä S.	2010	Spain	RT-PCR	EOC	miR-200a	FFPE	PFS	(M) 1.22 (0.57–2.58)	75% of positive cells
Leskelä S.	2010	Spain	RT-PCR	EOC	miR-200b	FFPE	PFS	(M) 1.35 (0.62–2.93)	
Leskelä S.	2010	Spain	RT-PCR	EOC	miR-200c	FFPE	PFS	(M) 2.24 (1.00–5.03)	
Leskelä S.	2010	Spain	RT-PCR	EOC	miR-141	FFPE	PFS	(M) 2.35 (0.98–5.59)	
Leskelä S.	2010	Spain	RT-PCR	EOC	miR-429	FFPE	PFS	(M) 2.10 (0.92–4.79)	
Leskelä S.	2010	Spain	RT-PCR	EOC	miR-429	FFPE	RFS	(M) 2.01 (1.11–3.66)	
Leskelä S.	2010	Spain	RT-PCR	EOC	miR-429	FFPE	OS	(M) 2.08 (1.03–4.20)	

Hu X.	2009	USA	RT-PCR	EOC	miR-200a	FFPE	OS	(U) 0.70 (0.03–14.29)	>11
Hu X.	2009	USA	RT-PCR	EOC	miR-200a	FFPE	PFS	(U) 0.64 (0.22–1.81)	

EOC: epithelial ovarian cancer; BC: breast cancer; NSCLC: nonsmall cell lung cancer; NMIBC: nonmuscle-invasive bladder cancer; GC: gastric cancer; CRC: colorectal cancer; OS: overall survival; DFS: disease-free survival; PFS: progression-free survival; RFS: recurrence- or relapse-free survival; HR: hazard ratio; CI: confidence interval; U: univariate analysis; M: multivariate analysis; ISH: in situ hybridization; RT-PCR: reverse transcription-polymerase chain reaction; FFPE: formalin-fixed and paraffin-embedded; TMA: tissue microarray; OS: overall survival; DFS: disease-free survival; PFS, progression-free survival; RFS: recurrence- or relapse-free survival.

**Table 3:** Stratified analysis of the high expression of the miR-200 family and overall survival.

Categories	Subgroups	Univariate analyses					Multivariate analyses				
		Number of datasets	HR (95% CI)	*P* value	*I* ^2^	Ph	Number of datasets	HR (95% CI)	*P* value	*I* ^2^	Ph
All		19	**1.32 (1.14–1.54)**	<0.001	77.50%	<0.001	24	**1.32 (1.16–1.49)**	<0.001	75.10%	<0.001

Patient source	Asia	10	**1.91 (1.26–2.92)**	0.003	80.10%	<0.001	13	**1.98 (1.34–2.90)**	0.001	78.20%	<0.001
	Europe	5	1.07 (0.95–1.21)	0.286	66.80%	<0.001	8	1.11 (0.99–1.24)	0.071	67.10%	<0.001
	North America	4	**3.81 (2.46–5.90)**	<0.001	0.00%	0.685	3	**2.37 (1.44–3.91)**	0.001	0.00%	0.457

Cancer type	EOC	7	**2.18 (1.23–3.86)**	0.008	79.90%	<0.001	7	**1.98 (1.03–3.80)**	0.039	81.80%	<0.001
	CRC	7	1.12 (0.96–1.31)	0.140	77.70%	<0.001	8	**1.15 (1.02–1.30)**	0.026	60.10%	0.001
	NSCLC	3	**3.36 (1.64–6.89)**	0.001	0.00%	0.411	6	**2.91 (1.99–4.26)**	<0.001	33.40%	0.185
	GC	1	0.94 (0.75–1.17)	0.565	1.90%	0.361	2	1.10 (0.72–1.68)	0.669	62.30%	0.047
	BC	1	**1.38 (1.11–1.71)**	0.003	/	/	1	1.46 (0.54–3.91)	0.454	75.10%	0.045

Test method	RT-PCR	16	1.64 (1.24–2.16)	0.001	75.30%	<0.001	19	**1.57 (1.23–1.99)**	<0.001	75.10%	<0.001
	ISH	1	**21.42 (7.54–60.83)**	<0.001	0.00%	0.996	1	**16.28 (6.28–42.24)**	<0.001	0.00%	0.995
	TaqMan	2	1.01 (0.95–1.08)	0.686	47.70%	0.046	4	1.07 (0.95–1.20)	0.249	58.80%	0.005

Sample source	FFPE	2	0.57 (0.29–1.10)	0.095	0.00%	0.890	5	**2.27 (1.56–3.32)**	<0.001	43.10%	0.135
	Tissue	5	**3.19 (1.19–8.52)**	0.021	84.40%	<0.001	9	**2.04 (1.13–3.68)**	0.017	80.70%	<0.001
	Blood	10	**1.34 (1.15–1.57)**	<0.001	79.00%	<0.001	9	**1.14 (1.02–1.28)**	0.019	68.30%	<0.001
	TMA	2	0.93 (0.75–1.16)	0.527	0.00%	0.519	1	0.94 (0.73–1.21)	0.649	40.90%	0.184

Sample size	≧100	11	**1.25 (1.06–1.47)**	0.007	78.90%	<0.001	14	**1.29 (1.11–1.49)**	0.001	71.60%	<0.001
	<100	8	**1.74 (1.10–2.75)**	0.018	68.50%	0.001	10	**1.84 (1.17–2.90)**	0.008	79.60%	<0.001

miR-200 component	miR-200a	7	1.14 (0.81–1.61)	0.438	64.80%	0.009	6	1.07 (0.72–1.59)	0.723	78.30%	<0.001
	miR-200b	6	1.38 (0.88–2.16)	0.166	82.10%	<0.001	6	1.36 (0.89–2.08)	0.158	76.70%	0.001
	miR-200c	11	**1.38 (1.01–1.89)**	0.040	82.40%	<0.001	10	**1.62 (1.12–2.33)**	0.010	79.30%	<0.001
	miR-141	7	**2.01 (1.26–3.21)**	0.003	83.50%	<0.001	7	1.24 (0.99–1.56)	0.060	68.00%	0.005
	miR-429	5	0.99 (0.73–1.34)	0.953	62.20%	0.032	8	**1.41 (1.01–1.98)**	0.043	70.30%	0.001

EOC: epithelial ovarian cancer; BC: breast cancer; NSCLC: nonsmall cell lung cancer; GC: gastric cancer; CRC: colorectal cancer; RT-PCR: reverse transcription-polymerase chain reaction; ISH: in situ hybridization; FFPE: formalin-fixed and paraffin-embedded; TMA: tissue microarray; HR: hazard ratio; CI: confidence interval; Ph: *P* value of the heterogeneity test.

## Data Availability

All data have been shared in the figures and tables.
